# Supplementary material for: Daily changes in phytoplankton lipidomes reveal mechanisms of energy storage in the open ocean
Source: Nat Commun. 2018 Dec 5;9:5179. doi: 10.1038/s41467-018-07346-z (PMC6281602; doi:10.1038/s41467-018-07346-z)
Supplement: Supplementary file 1 — Supplementary Information [file 41467_2018_7346_MOESM1_ESM.pdf]

Daily changes in phytoplankton lipidomes reveal mechanisms of energy storage in the open ocean

Kevin W. Becker[1], James R. Collins[1,2,†], Bryndan P. Durham[3], Ryan D. Groussman[3], Angelicque E. White[4], Helen F. Fredricks[1], Justin E. Ossolinski[1], Daniel J. Repeta[1], Paul Carini[5,+], E. Virginia Armbrust[3], and Benjamin A. S. Van Mooy[1]

[1]Department of Marine Chemistry and Geochemistry, Woods Hole Oceanographic Institution, Woods Hole, MA 02543, USA.

[2]Massachusetts Institute of Technology/Woods Hole Oceanographic Institution Joint Program in Oceanography, Woods Hole, MA 02543, United States

[3]School of Oceanography, University of Washington, Seattle, WA 98195, USA.

[4]College of Earth, Ocean, and Atmospheric Sciences, Oregon State University, Corvallis, OR 97331, USA.

[5]Department of Microbiology, Oregon State University, Corvallis, OR 97331, USA.

[†]Present address: School of Oceanography and eScience Institute, University of Washington, Seattle, WA 98195, USA

[+]Present address: Department of Soil, Water and Environmental Science, University of Arizona, Tucson, AZ 85721, USA

Supplementary Methods

Calculation of contribution of TAG production rates to oceanic primary production.

To estimate the global annual TAG synthesis rate in the subtropical gyres, we assumed the lower (Spring 2016) TAG production rates in Table 2 were representative of those throughout the year. The TAG production rate averaged over the sampling depth of 150 m was 0.22 mg C m^{-3} d^{-1}, and thus the vertically-integrated flux was 33 mg C m^{-2} d^{-1}. The total surface area of the subtropical gyres used for the calculation (1.97×10^8 km^2) was based on Sarmiento et al.[1].

Estimating increase in eukaryotic phytoplankton caloric content due to TAG increase during the day.

Platt and Irwin[2] showed that the caloric content of eukaryotic phytoplankton biomass by dry mass could be predicted to be within 20% based on a simple mixing equation based on the caloric value of the different classes of biochemicals and their respective dry mass percentages (cal mg^{-1} x 100):

$$Caloric\ value = 4.2\ cal\ mg^{-1}\ x\ \left(\%\frac{protein}{100}\right) + 4.2\ cal\ mg^{-1}\ x\ \left(\%\frac{carbohydrates}{100}\right) + 9.5\ cal\ mg^{-1}\ x\ \left(\%\frac{lipid}{100}\right) \quad \textbf{[1]}$$

Directly measuring the contribution of TAGs to phytoplankton biomass *in situ* is difficult because the contribution of phytoplankton to particulate organic carbon (POC) is highly variable. Assuming average contributions to dry mass of 10% lipids, 24% carbohydrate, and 18% protein (as determined by Platt and Irwin[2] for phytoplankton at dawn), we obtained a total caloric content of

2.7 cal mg^{-1}. If TAG concentrations increase 2.3-fold (Fig. 1b) during the day to 23% lipids while the fraction of other biochemical classes remain the same, then this yields a caloric content of 3.9 cal mg^{-1} at dusk, which is a 42% increase per unit dry mass.

Modeling the loss of TAGs at night due to cellular respiration.

An inverse modeling approach was taken to assess the relative contribution of cell mortality versus intracellular respiration on the observed decrease of TAGs each night. The first step was to apply a simple mass-balance model for changes in concentration of the membrane lipid DGCC, which is a proxy for eukaryotic nanoplankton cell abundance and, thus, a tracer for growth and mortality:

$$^{d[DGCC]}/_{dt} = \mu[DGCC] - m[DGCC] \qquad \textbf{[2]}$$

The mortality rate, m, was held constant throughout the day and night, based on empirical evidence that grazing by vertically migrating zooplankton consumes < 20% of primary production at Station ALOHA[3,4]. We allowed the growth rate, μ, to vary as a linear function of solar irradiance, I, and yield, φ_g.

$$\mu = \varphi_g \times I \qquad \textbf{[3]}$$

A sine wave function was used to simulate diel changes in I as a function of the time of day, h, according to:

$$I(h) = \begin{cases} 0, 0:00 \leq h < 6:00 \\ \sin\big((h - 6:00) \times 15^{\mathrm{o}}\big), 6:00 \leq h < 18:00 \\ 0, 18:00 \leq h \leq 24:00 \end{cases} \qquad \textbf{[4]}$$

Given an initial [DGCC] of 10 ng L^{-1} at 6:00, φ_g and m were optimized to reproduce the fold-change in [DGCC] observed over the course of the day. We then developed a second mass-balance model to describe the change in TAG concentration,

$$d[TAG]/_{dt} = \mu[TAG] + \sigma[TAG] - m[TAG] - r[TAG] \quad \textbf{[5]}$$

where cell growth, μ, was simulated according to Eq. 3 using the optimal value of φ_g determined above, and the rate of cell mortality, m, was assumed to be the same as in Eq. 2. The additional loss term r[TAG] accounted for the respiration of TAGs during the night at rate r. The additional source term σ[TAG] was necessary to account for the irradiance-mediated accumulation TAGs during the day at rate σ based on the observation that TAGs are accumulated in the cell at rates in excess of that predicted by cell growth alone.

Similar to μ, the parameter σ was modeled as a linear function of I and an independent yield for TAGs, φ_s.

$$\sigma = \varphi_s \times I \quad \textbf{[6]}$$

The nighttime respiration rate r was defined according to

$$r = \begin{cases} \rho, 0{:}00 \le h < 6{:}00 \\ 0, 6{:}00 \le h < 18{:}00 \\ \rho, 18{:}00 \le h \le 24{:}00 \end{cases} \quad \textbf{[7]}$$

where ρ was the night respiration factor. Optimized parameters (Supplementary Table 1) and an initial [TAG] of 1,500 ng L^{-1} at 6:00 reproduced the diel cycles with a remarkably high level of skill given the simplicity of the model (Supplementary Figure 11); residuals averaged 6%.

The conclusion from this model is that the mortality rate (0.0192 h^{-1}) is smaller than cellular respiration rate of TAGs (0.0473 h^{-1}). Thus, the model predicted that 29% of the daily TAG decrease is due to mortality process while 71% is due to intracellular respiration.

Supplementary Figures

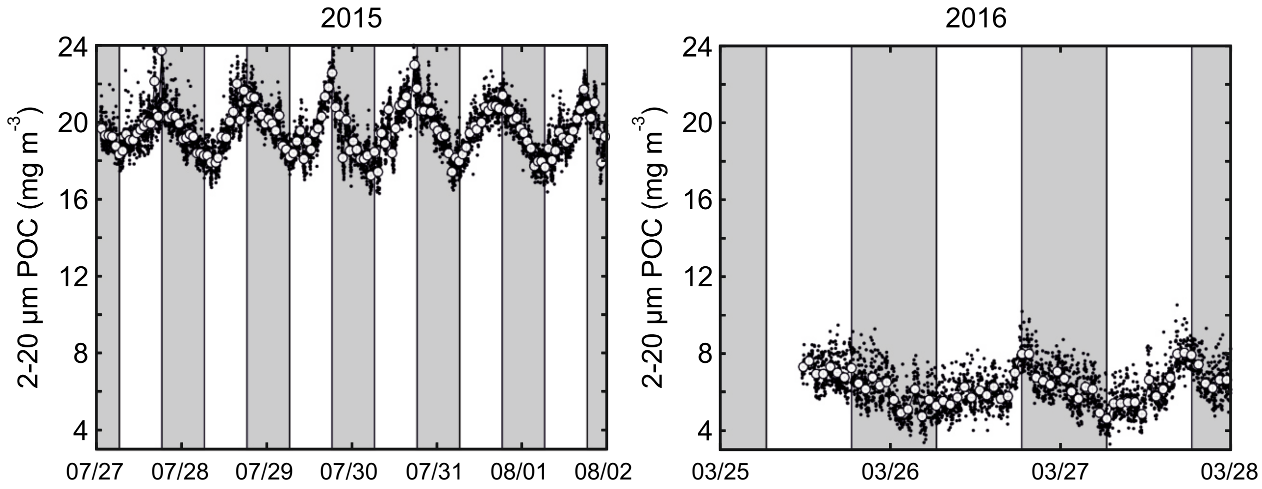

Supplementary Figure 1. Diel variability in particulate organic carbon (POC) content. a 2-20 µm POC concentration during the sampling perio d insummer 2015 and **b**, spring 2016. Grey bars indicate night.

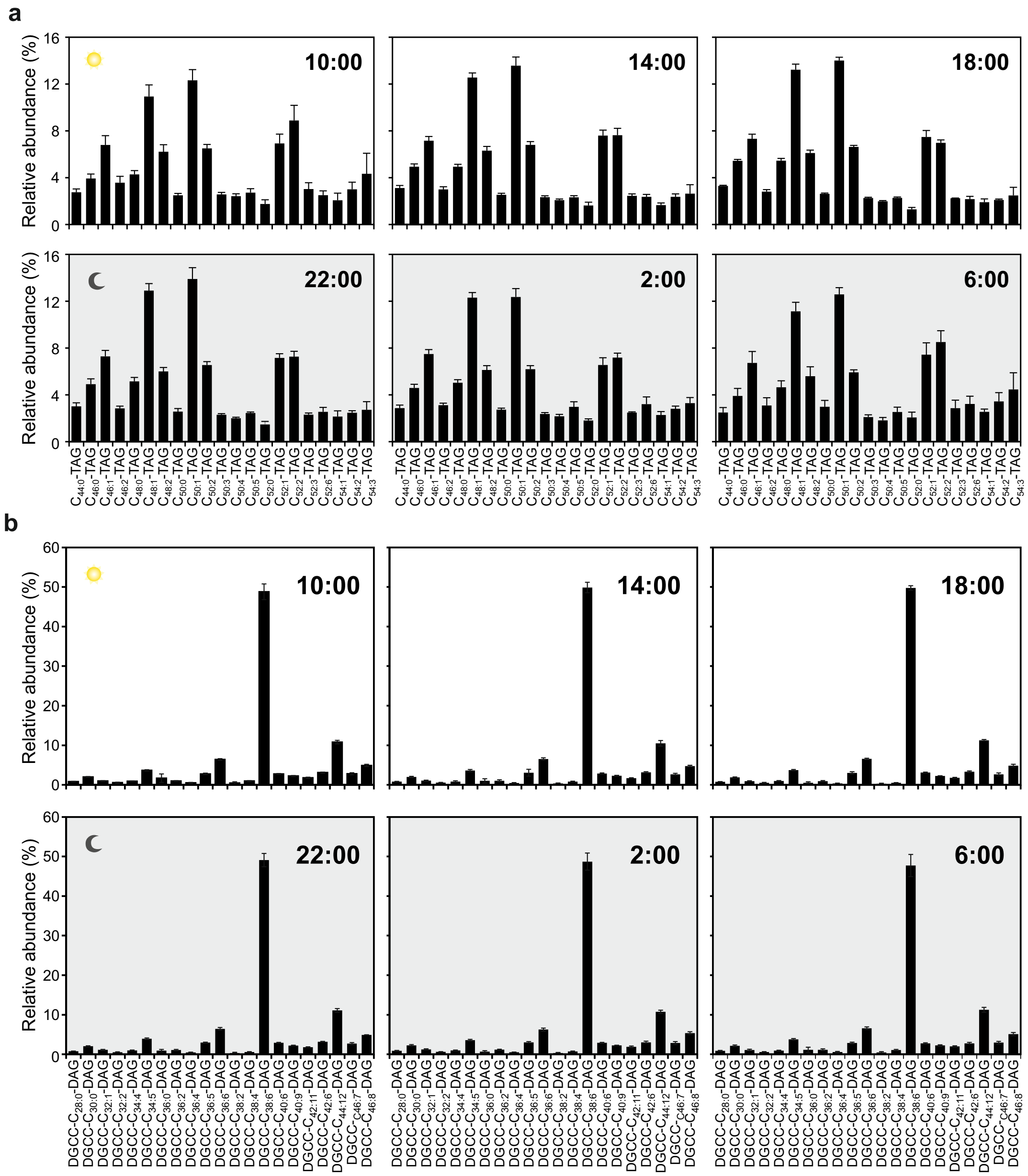

Supplementary Figure 2. Triacylglycerol and betaine lipid composition. Histograms of relative abundances of triacylglycerols (TAG) (**a**) and the betaine lipids dacylglycerylcarboxy -*N*-hydroxymethyl-cholines (DGCC) diacylglycerols (DAG) (**b**) averaged for time of day in samples collected in Jul/August 2015 during *R/V* Kilo Moana cruise KM1513 at 15 m water depth. Bars are mean ± SD (n ≥ 6). White background in graphs indicates daylight hours and gray background dark hours.

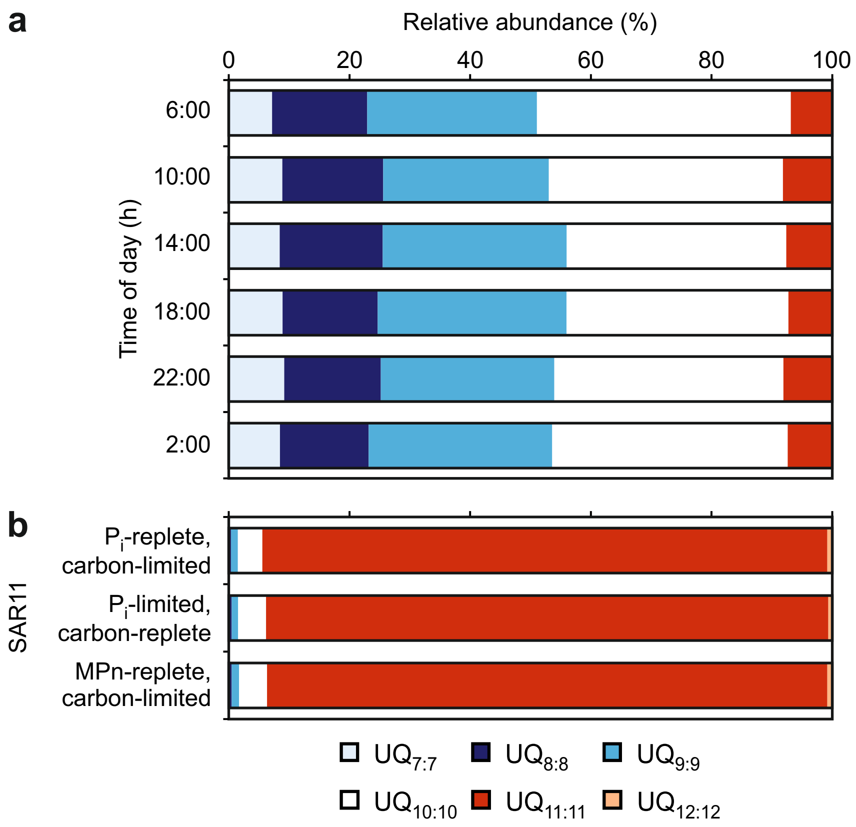

Supplementary Figure3 . Ubiquinone composition. a, Relative distribution of ubiquinones (UQs) in the diel samples (averaged for time of day) collected during *R/V* Kilo Moana cruise KM1513 at 15 m water depth and **b**, in SAR11 cultures harvested during exponential growth (T_{48}; cf. Carini et al.[5]). Quinone nomenclature is after Elling et al.[6].

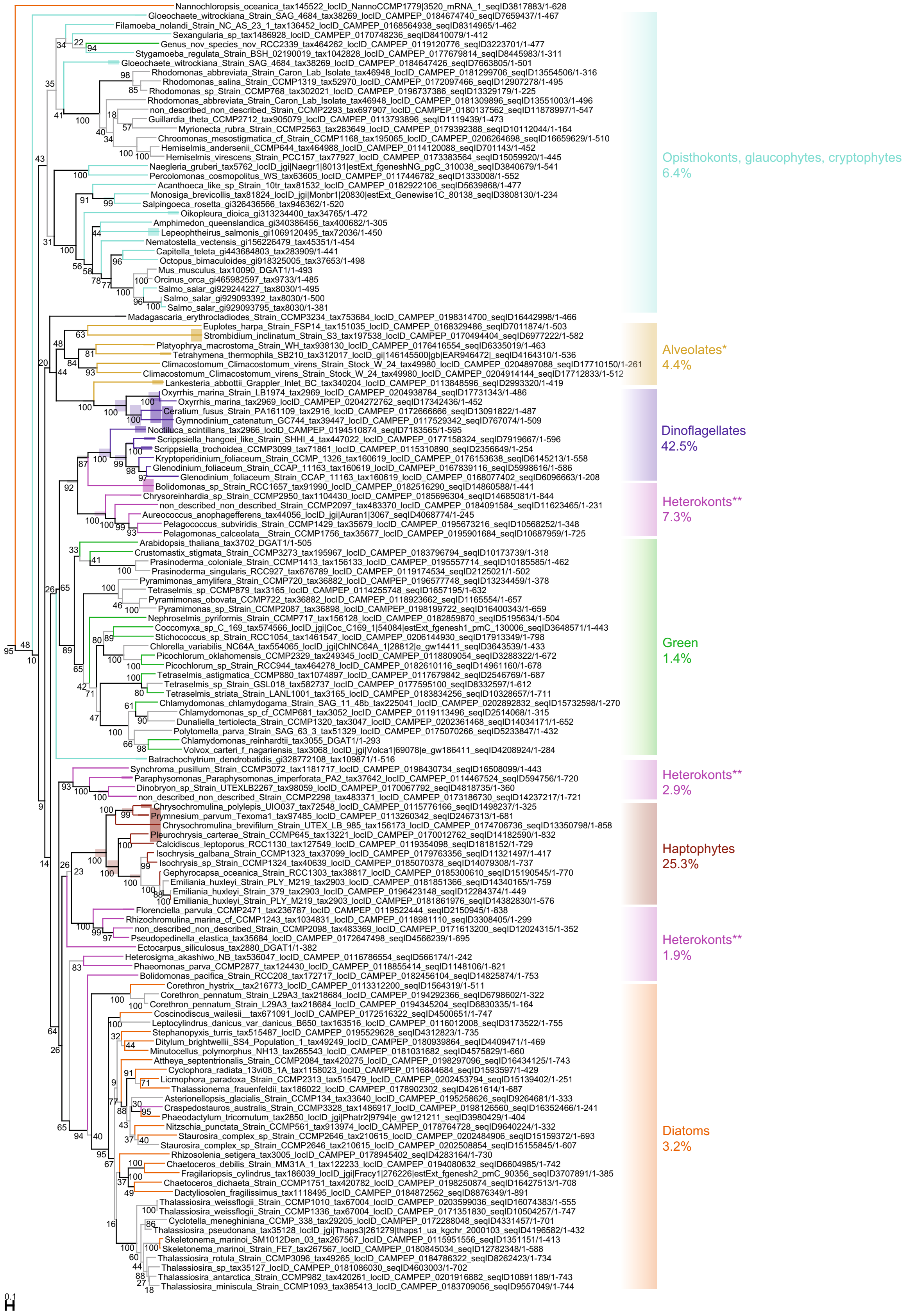

Nannochloropsis_oceanica_tax145522_locID_NannoCCMP1779|3520_mRNA_1_seqID3817883/1-628
Gloeochaete_witrockiana_Strain_SAG_4684_tax38269_locID_CAMPEP_0184674740_seqID7659437/1-467
Filamoeba_nolandi_Strain_NC_AS_23_1_tax136452_locID_CAMPEP_0168564938_seqID8314965/1-462
Sexangularia_sp_tax1486928_locID_CAMPEP_0170748236_seqID8410079/1-412
Genus_nov_species_nov_RCC2339_tax464262_locID_CAMPEP_0119120776_seqID3223701/1-477
Stygamoeba_regulata_Strain_BSH_02190019_tax1042828_locID_CAMPEP_0177679814_seqID8445983/1-311
Gloeochaete_witrockiana_Strain_SAG_4684_tax38269_locID_CAMPEP_0184647426_seqID7663805/1-501
Rhodomonas_abbreviata_Strain_Caron_Lab_Isolate_tax46948_locID_CAMPEP_0181299706_seqID13554506/1-316
Rhodomonas_salina_Strain_CCMP1319_tax52970_locID_CAMPEP_0172097466_seqID12907278/1-495
Rhodomonas_sp_Strain_CCMP768_tax302021_locID_CAMPEP_0196737386_seqID13329179/1-225
Rhodomonas_abbreviata_Strain_Caron_Lab_Isolate_tax46948_locID_CAMPEP_0181309896_seqID13551003/1-496
non_described_non_described_Strain_CCMP2293_tax697907_locID_CAMPEP_0180137562_seqID11878997/1-547
Guillardia_theta_CCMP2712_tax905079_locID_CAMPEP_0113793896_seqID1119439/1-473
Myrionecta_rubra_Strain_CCMP2563_tax283649_locID_CAMPEP_0179392388_seqID10112044/1-164
Chroomonas_mesostigmatica_cf_Strain_CCMP1168_tax195065_locID_CAMPEP_0206264698_seqID16659629/1-510
Hemiselmis_andersenii_CCMP644_tax464988_locID_CAMPEP_0114120088_seqID701143/1-452
Hemiselmis_virescens_Strain_PCC157_tax77927_locID_CAMPEP_0173383564_seqID15059920/1-445
Naegleria_gruberi_tax5762_locID_jgi|Naegr1|80131|estExt_fgeneshNG_pgC_310038_seqID3840679/1-541
Percolomonas_cosmopolitus_WS_tax63605_locID_CAMPEP_0117446782_seqID1333008/1-552
Acanthoeca_like_sp_Strain_10tr_tax81532_locID_CAMPEP_0182922106_seqID5639868/1-477
Monosiga_brevicollis_tax81824_locID_jgi|Monbr1|20830|estExt_Genewise1C_80138_seqID3808130/1-234
Salpingoeca_rosetta_gi326436566_tax946362/1-520
Oikopleura_dioica_gi313234400_tax34765/1-472
Amphimedon_queenslandica_gi340386456_tax400682/1-305
Lepeophtheirus_salmonis_gi1069120495_tax72036/1-450
Nematostella_vectensis_gi156226479_tax45351/1-454
Capitella_teleta_gi443684803_tax283909/1-441
Octopus_bimaculoides_gi918325005_tax37653/1-498
Mus_musculus_tax10090_DGAT1/1-493
Orcinus_orca_gi465982597_tax9733/1-485
Salmo_salar_gi929244227_tax8030/1-495
Salmo_salar_gi929093392_tax8030/1-500
Salmo_salar_gi929093795_tax8030/1-381
Madagascaria_erythrocladiodes_Strain_CCMP3234_tax753684_locID_CAMPEP_0198314700_seqID16442998/1-466
Euplotes_harpa_Strain_FSP14_tax151035_locID_CAMPEP_0168329486_seqID7011874/1-503
Strombidium_inclinatum_Strain_S3_tax197538_locID_CAMPEP_0170494404_seqID6977222/1-582
Platyophrya_macrostoma_Strain_WH_tax938130_locID_CAMPEP_0176416554_seqID6335019/1-463
Tetrahymena_thermophila_SB210_tax312017_locID_gi|146145500|gb|EAR946472|_seqID4164310/1-536
Climacostomum_Climacostomum_virens_Strain_Stock_W_24_tax49980_locID_CAMPEP_0204897088_seqID17710150/1-261
Climacostomum_Climacostomum_virens_Strain_Stock_W_24_tax49980_locID_CAMPEP_0204914144_seqID17712833/1-512
Lankesteria_abbottii_Grappler_Inlet_BC_tax340204_locID_CAMPEP_0113848596_seqID2993320/1-419
Oxyrrhis_marina_Strain_LB1974_tax2969_locID_CAMPEP_0204938784_seqID17731343/1-486
Oxyrrhis_marina_tax2969_locID_CAMPEP_0204272762_seqID17342436/1-452
Ceratium_fusus_Strain_PA161109_tax2916_locID_CAMPEP_0172666666_seqID13091822/1-487
Gymnodinium_catenatum_GC744_tax39447_locID_CAMPEP_0117529342_seqID767074/1-509
Noctiluca_scintillans_tax2966_locID_CAMPEP_0194510874_seqID7183565/1-595
Scrippsiella_hangoei_like_Strain_SHHI_4_tax447022_locID_CAMPEP_0177158324_seqID7919667/1-596
Scrippsiella_trochoidea_CCMP3099_tax71861_locID_CAMPEP_0115310890_seqID2356649/1-254
Kryptoperidinium_foliaceum_Strain_CCMP_1326_tax160619_locID_CAMPEP_0176153638_seqID6145213/1-558
Glenodinium_foliaceum_Strain_CCAP_11163_tax160619_locID_CAMPEP_0167839116_seqID5998616/1-586
Glenodinium_foliaceum_Strain_CCAP_11163_tax160619_locID_CAMPEP_0168077402_seqID6096663/1-208
Bolidomonas_sp_Strain_RCC1657_tax91990_locID_CAMPEP_0182516290_seqID14860588/1-441
Chrysoreinhardia_sp_Strain_CCMP2950_tax1104430_locID_CAMPEP_0185696304_seqID14685081/1-844
non_described_non_described_Strain_CCMP2097_tax483370_locID_CAMPEP_0184091584_seqID11623465/1-231
Aureococcus_anophagefferens_tax44056_locID_jgi|Auran1|3067_seqID4068774/1-245
Pelagococcus_subviridis_Strain_CCMP1429_tax35679_locID_CAMPEP_0195673216_seqID10568252/1-348
Pelagomonas_calceolata__Strain_CCMP1756_tax35677_locID_CAMPEP_0195901684_seqID10687959/1-725
Arabidopsis_thaliana_tax3702_DGAT1/1-505
Crustomastix_stigmata_Strain_CCMP3273_tax195967_locID_CAMPEP_0183796794_seqID10173739/1-318
Prasinoderma_coloniale_Strain_CCMP1413_tax156133_locID_CAMPEP_0195557714_seqID10185585/1-462
Prasinoderma_singularis_RCC927_tax676789_locID_CAMPEP_0119174534_seqID2125021/1-502
Pyramimonas_amylifera_Strain_CCMP720_tax36882_locID_CAMPEP_0196577748_seqID13234459/1-378
Tetraselmis_sp_CCMP879_tax3165_locID_CAMPEP_0114255748_seqID1657195/1-632
Pyramimonas_obovata_CCMP722_tax36882_locID_CAMPEP_0118923662_seqID1165554/1-657
Pyramimonas_sp_Strain_CCMP2087_tax36898_locID_CAMPEP_0198199722_seqID16400343/1-659
Nephroselmis_pyriformis_Strain_CCMP717_tax156128_locID_CAMPEP_0182859870_seqID5195634/1-504
Coccomyxa_sp_C_169_tax574566_locID_jgi|Coc_C169_1|54084|estExt_fgenesh1_pmC_130006_seqID3648571/1-443
Stichococcus_sp_Strain_RCC1054_tax1461547_locID_CAMPEP_0206144930_seqID17913349/1-798
Chlorella_variabilis_NC64A_tax554065_locID_jgi|ChlNC64A_1|28812|e_gw14411_seqID3643539/1-433
Picochlorum_oklahomensis_CCMP2329_tax249345_locID_CAMPEP_0118809054_seqID3288322/1-672
Picochlorum_sp_Strain_RCC944_tax464278_locID_CAMPEP_0182610116_seqID14961160/1-678
Tetraselmis_astigmatica_CCMP880_tax1074897_locID_CAMPEP_0117679842_seqID2546769/1-687
Tetraselmis_sp_Strain_GSL018_tax582737_locID_CAMPEP_0177595100_seqID8332597/1-612
Tetraselmis_striata_Strain_LANL1001_tax3165_locID_CAMPEP_0183834256_seqID10328657/1-711
Chlamydomonas_chlamydogama_Strain_SAG_11_48b_tax225041_locID_CAMPEP_0202892832_seqID15732598/1-270
Chlamydomonas_sp_cf_CCMP681_tax3052_locID_CAMPEP_0119113496_seqID2514068/1-315
Dunaliella_tertiolecta_Strain_CCMP1320_tax3047_locID_CAMPEP_0202361468_seqID14034171/1-652
Polytomella_parva_Strain_SAG_63_3_tax51329_locID_CAMPEP_0175070266_seqID5233847/1-432
Chlamydomonas_reinhardtii_tax3055_DGAT1/1-293
Volvox_carteri_f_nagariensis_tax3068_locID_jgi|Volca1|69078|e_gw186411_seqID4208924/1-284
Batrachochytrium_dendrobatidis_gi328772108_tax109871/1-516
Synchroma_pusillum_Strain_CCMP3072_tax1181717_locID_CAMPEP_0198430734_seqID16508099/1-443
Paraphysomonas_Paraphysomonas_imperforata_PA2_tax37642_locID_CAMPEP_0114467524_seqID594756/1-720
Dinobryon_sp_Strain_UTEXLB2267_tax98059_locID_CAMPEP_0170067792_seqID4818735/1-360
non_described_non_described_Strain_CCMP2298_tax483371_locID_CAMPEP_0173186730_seqID14237217/1-721
Chrysochromulina_polylepis_UIO037_tax72548_locID_CAMPEP_0115776166_seqID1498237/1-325
Prymnesium_parvum_Texoma1_tax97485_locID_CAMPEP_0113260342_seqID2467313/1-681
Chrysochromulina_brevifilum_Strain_UTEX_LB_985_tax156173_locID_CAMPEP_0174706736_seqID13350798/1-858
Pleurochrysis_carterae_Strain_CCMP645_tax13221_locID_CAMPEP_0170012762_seqID14182590/1-832
Calcidiscus_leptoporus_RCC1130_tax127549_locID_CAMPEP_0119354098_seqID1818152/1-729
Isochrysis_galbana_Strain_CCMP1323_tax37099_locID_CAMPEP_0179763356_seqID11321497/1-417
Isochrysis_sp_Strain_CCMP1324_tax40639_locID_CAMPEP_0185070378_seqID14079308/1-737
Gephyrocapsa_oceanica_Strain_RCC1303_tax38817_locID_CAMPEP_0185300610_seqID15190545/1-770
Emiliania_huxleyi_Strain_PLY_M219_tax2903_locID_CAMPEP_0181851366_seqID14340165/1-759
Emiliania_huxleyi_Strain_379_tax2903_locID_CAMPEP_0196423148_seqID12284374/1-449
Emiliania_huxleyi_Strain_PLY_M219_tax2903_locID_CAMPEP_0181861976_seqID14382830/1-576
Florenciella_parvula_CCMP2471_tax236787_locID_CAMPEP_0119522444_seqID2150945/1-838
Rhizochromulina_marina_cf_CCMP1243_tax1034831_locID_CAMPEP_0118981110_seqID3308405/1-299
non_described_non_described_Strain_CCMP2098_tax483369_locID_CAMPEP_0171613200_seqID12024315/1-352
Pseudopedinella_elastica_tax35684_locID_CAMPEP_0172647498_seqID4566239/1-695
Ectocarpus_siliculosus_tax2880_DGAT1/1-382
Heterosigma_akashiwo_NB_tax536047_locID_CAMPEP_0116786554_seqID566174/1-242
Phaeomonas_parva_CCMP2877_tax124430_locID_CAMPEP_0118855414_seqID1148106/1-821
Bolidomonas_pacifica_Strain_RCC208_tax172717_locID_CAMPEP_0182456104_seqID14825874/1-753
Corethron_hystrix__tax216773_locID_CAMPEP_0113312200_seqID1564319/1-511
Corethron_pennatum_Strain_L29A3_tax218684_locID_CAMPEP_0194292366_seqID6798602/1-322
Corethron_pennatum_Strain_L29A3_tax218684_locID_CAMPEP_0194345204_seqID6830335/1-164
Coscinodiscus_wailesii__tax671091_locID_CAMPEP_0172516322_seqID4500651/1-747
Leptocylindrus_danicus_var_danicus_B650_tax163516_locID_CAMPEP_0116012008_seqID3173522/1-755
Stephanopyxis_turris_tax515487_locID_CAMPEP_0195529628_seqID4312823/1-735
Ditylum_brightwellii_SS4_Population_1_tax49249_locID_CAMPEP_0180939864_seqID4409471/1-469
Minutocellus_polymorphus_NH13_tax265543_locID_CAMPEP_0181031682_seqID4575829/1-660
Attheya_septentrionalis_Strain_CCMP2084_tax420275_locID_CAMPEP_0198297096_seqID16434125/1-743
Cyclophora_radiata_13vi08_1A_tax1158023_locID_CAMPEP_0116844684_seqID1593597/1-429
Licmophora_paradoxa_Strain_CCMP2313_tax515479_locID_CAMPEP_0202453794_seqID15139402/1-251
Thalassionema_frauenfeldii_tax186022_locID_CAMPEP_0178902302_seqID4261614/1-687
Asterionellopsis_glacialis_Strain_CCMP134_tax33640_locID_CAMPEP_0195258626_seqID9264681/1-333
Craspedostauros_australis_Strain_CCMP3328_tax1486917_locID_CAMPEP_0198126560_seqID16352466/1-241
Phaeodactylum_tricornutum_tax2850_locID_jgi|Phatr2|9794|e_gw121211_seqID3980429/1-404
Nitzschia_punctata_Strain_CCMP561_tax913974_locID_CAMPEP_0178764728_seqID9640224/1-332
Staurosira_complex_sp_Strain_CCMP2646_tax210615_locID_CAMPEP_0202484906_seqID15159372/1-693
Staurosira_complex_sp_Strain_CCMP2646_tax210615_locID_CAMPEP_0202508854_seqID15155845/1-607
Rhizosolenia_setigera_tax3005_locID_CAMPEP_0178945402_seqID4283164/1-730
Chaetoceros_debilis_Strain_MM31A_1_tax122233_locID_CAMPEP_0194080632_seqID6604985/1-742
Fragilariopsis_cylindrus_tax186039_locID_jgi|Fracy1|276226|estExt_fgenesh2_pmC_90356_seqID3707891/1-385
Chaetoceros_dichaeta_Strain_CCMP1751_tax420782_locID_CAMPEP_0198250874_seqID16427513/1-708
Dactyliosolen_fragilissimus_tax1118495_locID_CAMPEP_0184872562_seqID8876349/1-891
Thalassiosira_weissflogii_Strain_CCMP1010_tax67004_locID_CAMPEP_0203599036_seqID16074383/1-555
Thalassiosira_weissflogii_Strain_CCMP1336_tax67004_locID_CAMPEP_0171351830_seqID10504257/1-747
Cyclotella_meneghiniana_CCMP_338_tax29205_locID_CAMPEP_0172288048_seqID4331457/1-701
Thalassiosira_pseudonana_tax35128_locID_jgi|Thaps3|261279|thaps1_ua_kgchr_2000103_seqID4196582/1-432
Skeletonema_marinoi_SM1012Den_03_tax267567_locID_CAMPEP_0115951556_seqID1351151/1-413
Skeletonema_marinoi_Strain_FE7_tax267567_locID_CAMPEP_0180845034_seqID12782348/1-588
Thalassiosira_rotula_Strain_CCMP3096_tax49265_locID_CAMPEP_0184786322_seqID8262423/1-734
Thalassiosira_sp_tax35127_locID_CAMPEP_0181086030_seqID4603003/1-702
Thalassiosira_antarctica_Strain_CCMP982_tax420261_locID_CAMPEP_0201916882_seqID10891189/1-743
Thalassiosira_miniscula_Strain_CCMP1093_tax385413_locID_CAMPEP_0183709056_seqID9557049/1-744
Opisthokonts, glaucophytes, cryptophytes
6.4%
Alveolates*
4.4%
Dinoflagellates
42.5%
Heterokonts**
7.3%
Green
1.4%
Heterokonts**
2.9%
Haptophytes
25.3%
Heterokonts**
1.9%
Diatoms
3.2%
0.1

Supplementary Figure 4. Phylogeny of diacylglycerol transferase 1 (DGAT1) transcript abundance in the North Pacific Subtropical gyre. Maximum-likelihood phylogenetic tree for DGAT1 constructed from publicly available marine genomes and transcriptomes with environmental metatranscriptome read sequences aligned using pplacer[7]. External branches with read placement are colored by taxonomy. Internal branches with read placement are black. Gray branches indicate no read placement. Branch widths indicate the number of aligned reads and are colored by taxonomic signature (if assigned). Reads with poor placement (pendant length >1.0) were removed. Clades are colored with respect to their dominant taxonomic signature (*not including dinoflagellates (Dinophyceae), **not including diatoms (Bacillariophyta)) and the percentage of aligned reads is given. Outgroups include sterol acyltransferase (SAT) sequences. Scale bar indicates the number of substitutions per site. Bootstrap values are shown at nodes (n=100).

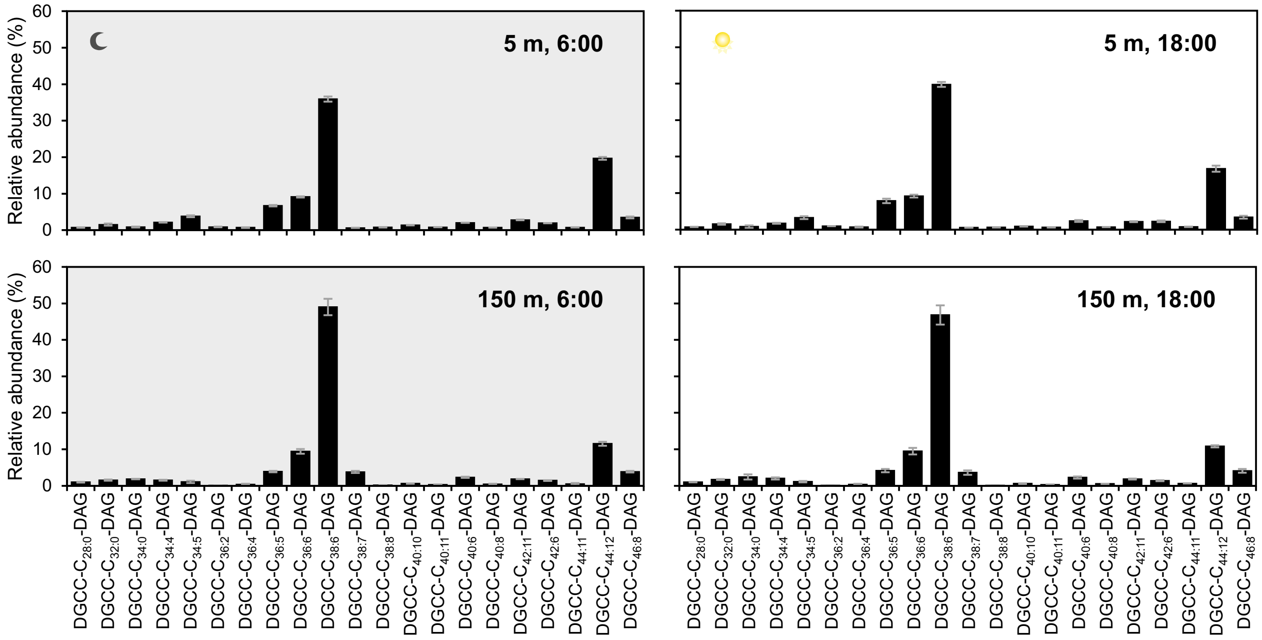

Supplementary Figure 5. Betaine lipid composition. Histograms of relative abundances of the betaine lipids diacylglycerylcarboxy-*N*-hydroxymethyl-cholines (DGCCs) diacylglycerol (DAG) averaged for time of day in samples collected in March 2016 during *R/V* Kilo Moana cruise KM1605 at 5 m and 150 m water depth. Bars are mean ± SD (n ≥ 3). White background in graphs indicates daylight hours and gray background dark hours.

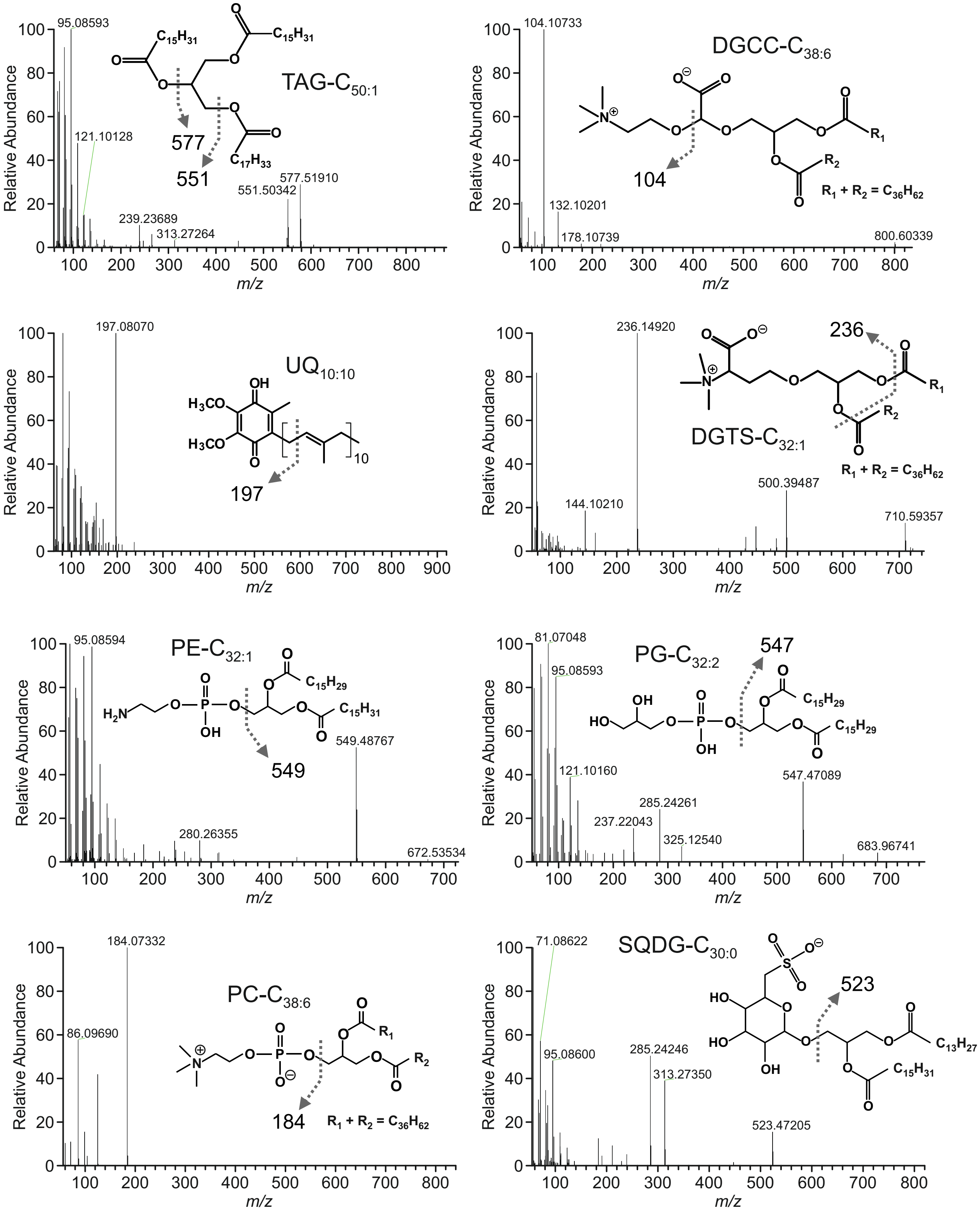

TAG-C50:1
95.08593
121.10128
239.23689
313.27264
551.50342
577.51910
577
551
DGCC-C38:6
104.10733
132.10201
178.10739
800.60339
104
R1 + R2 = C36H62
UQ10:10
197.08070
197
DGTS-C32:1
236.14920
144.10210
500.39487
710.59357
236
R1 + R2 = C36H62
PE-C32:1
95.08594
280.26355
549.48767
672.53534
549
PG-C32:2
81.07048
95.08593
121.10160
237.22043
285.24261
325.12540
547.47089
683.96741
547
PC-C38:6
184.07332
86.09690
184
R1 + R2 = C36H62
SQDG-C30:0
71.08622
95.08600
285.24246
313.27350
523.47205
523
Relative Abundance
m/z

Supplementary Figure 6. Mass spectra of representative lipids used to confirm identification by the LOBSTAHS software. MS^2 spectra of $[M+NH_4]^+$ at *m/z* 850.78619 of TAG-$C_{50:1}$, $[M+H]^+$ at *m/z* 800.60339 of DGCC-$C_{38:6}$, $[M+NH_4]^+$ at *m/z* 880.71777 of $UQ_{10:10}$, $[M+H]^+$ at *m/z* 710.59357 of DGTS/DGTA-$C_{38:6}$, $[M+H]^+$ at *m/z* 690.50745 of PE-$C_{32:1}$, $[M+NH_4]^+$ at *m/z* 736.51294 of PG-$C_{32:2}$, $[M+H]^+$ at *m/z* 806.56946 of PC-$C_{38:6}$, $[M+NH_4]^+$ at *m/z* 784.52399 of SQDG-$C_{30:0}$ in a representative sample from 15 m water depth collected during *R/V* Kilo Moana cruise KM1513 in July/August 2015. Fragmentation of the betaine lipids DGTS and DGTA results in the same fragment ions and they can thus not be distinguished with the used method. TAG, triacylglycerol; DGCC, diacylglycerylcarboxy-*N*-hydroxymethyl-choline, UQ, ubiquione; DGTS, diacylglyceryl trimethyl homoserine; DGTA, diacylglyceryl hydroxymethyl trimethyl-β-alanine; PE, phosphoethanolamine; PG; phosphatidylglycerol; PC, phosphochilone; SQDG, sulfoquinovosyl diacylglycerol.

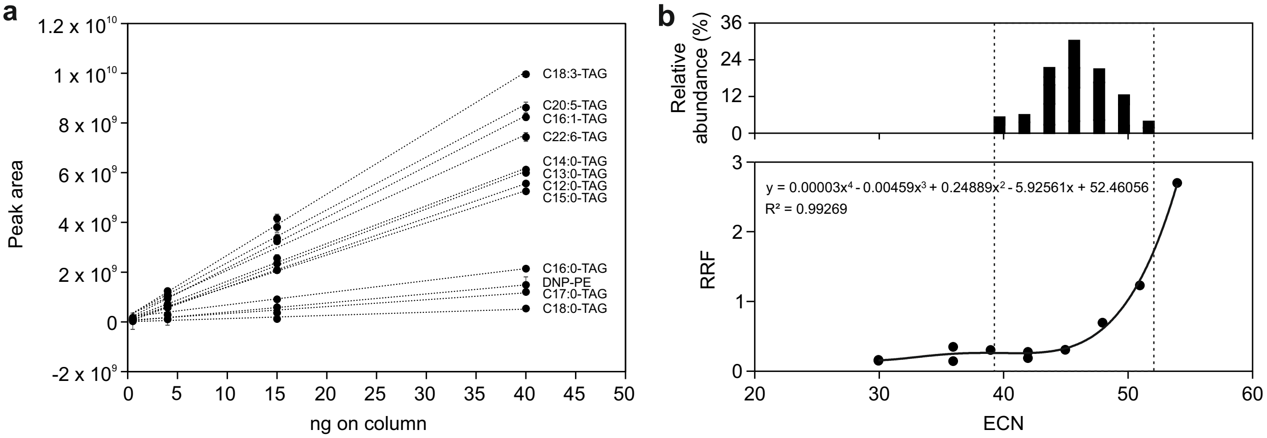

Supplementary Figure 7. Triacylglycerol calibration. a, Calibration curve of a triacylglycerol (TAG) standard mix and the internal standard DNP-PE (2,4-dinitrophenyl phosphatidylethanolamine) measured in triplicate by HPLC-ESI-MS. **b**, Lower panel: Dependence of relative response factor (RRF) for each TAG molecule on equivalent carbon number. Upper panel: Relative abundance of TAGs, classified by equivalent carbon number (ECN), in samples from the summer 2015 cruise. The relative abundances presented here were determined in samples collected at 10:00 h local time. Error bars represent standard deviation of triplicates.

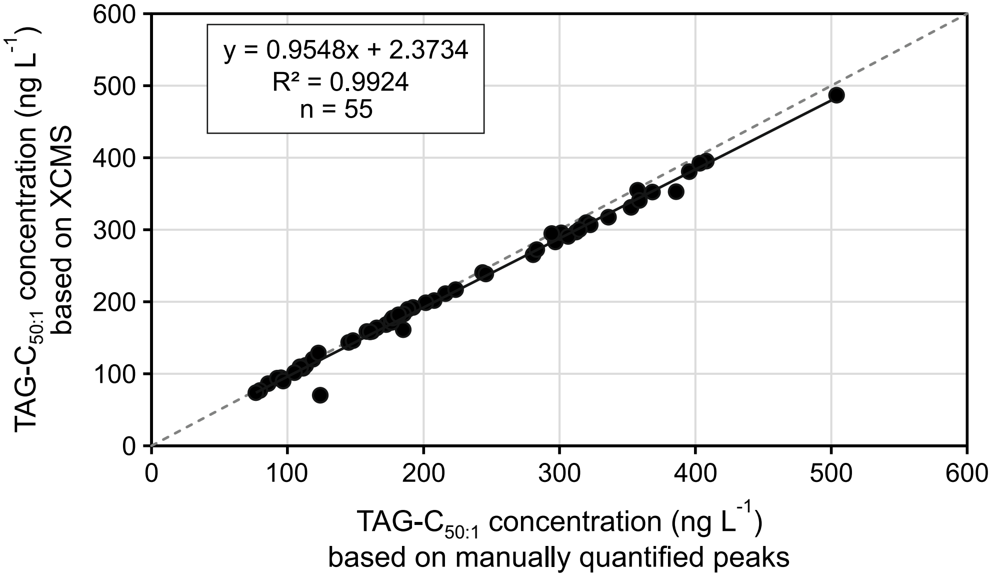

Supplementary Figure 8. Validation of peak detection by XCMS. Cross plot of concentrations of a triacylglycerol (TAG-$C_{50:1}$) in 55 samples based on peak areas obtained from the XCMS software and manually integrated peaks. Dashed line indicates the 1:1 line.

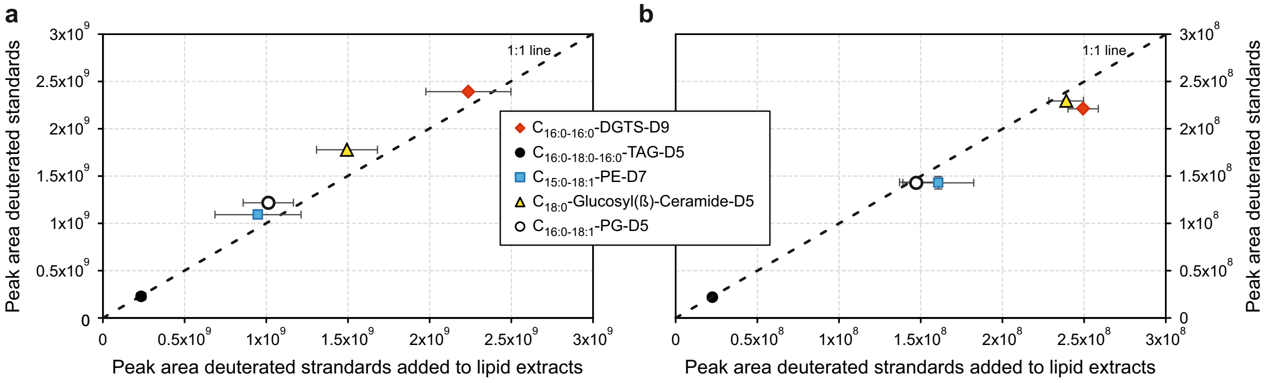

Supplementary Figure 9. Signal response comparisons for deuterated standards. Cross plots of peak areas of deuterated standards versus deuterated standards added to lipid extracts from samples collected in spring 2016 (**a**) and summer 2015 (**b**). Horizontal error bars represent the standard deviation of averaged values with n = 13 for **a** and n = 17 for **b**. PE, phosphatidylethanolamine; DGTS, diacylglyceryltrimethylhomoserine; PG, phosphatidylglycerol; TAG, triacylglycerol.

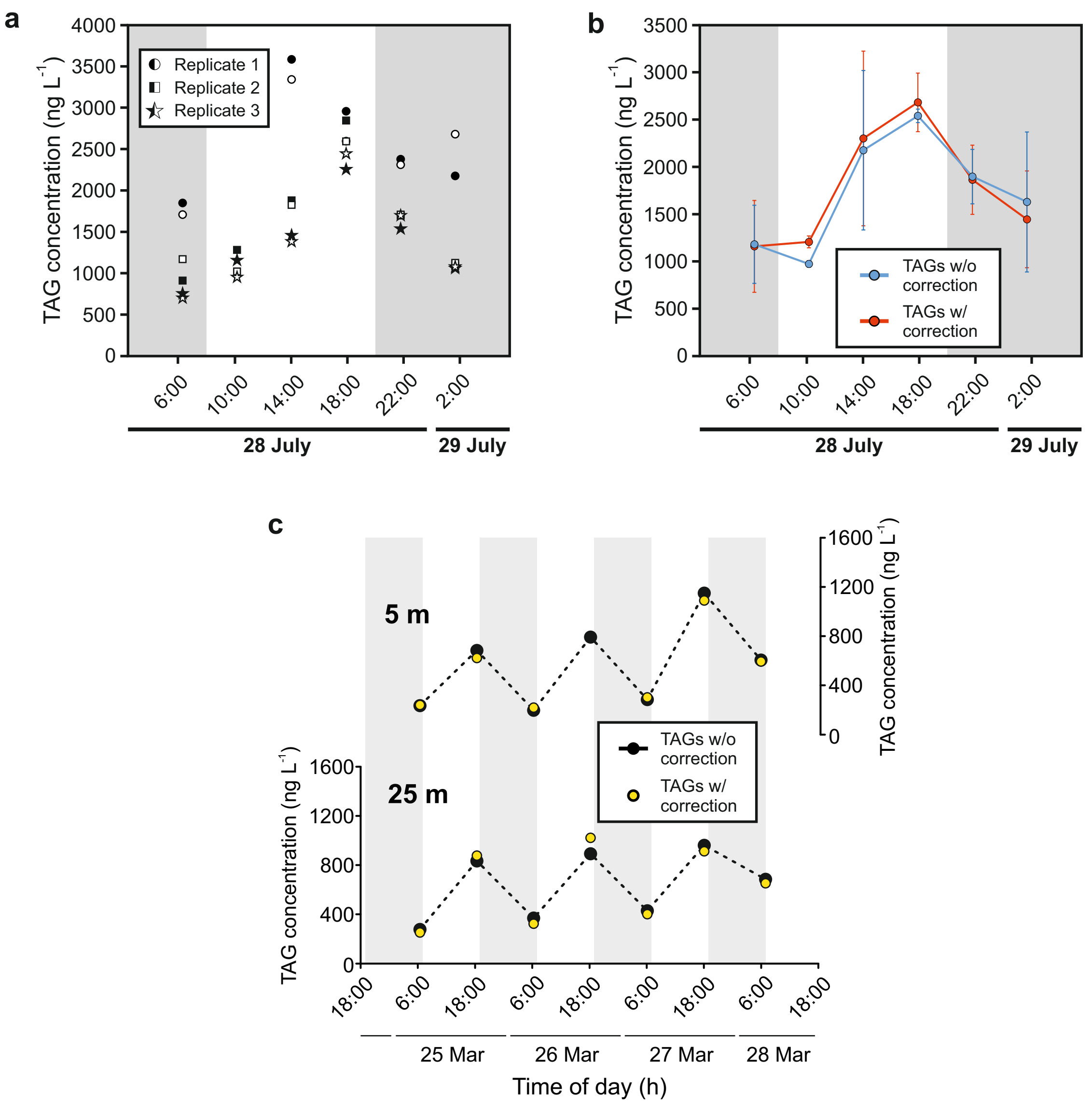

Supplementary Figure 10. Effects of ion suppression on triacylglycerol concentrations from deuterated standard addition experiments. a, Triacylglaycerol (TAG) concentration of triplicate samples from July 2015 before (closed symbols) and after (open symbols) correction for ion suppression. **b**. Comparison of TAG profiles for one 24 h period corrected (red) and uncorrected (blue) for ion suppression. **c**, TAG concentration in samples from March 2016 before (black circles) and after (yellow circles) correction for ion suppression. Error bars in **b** represent the standard deviation of triplicates.

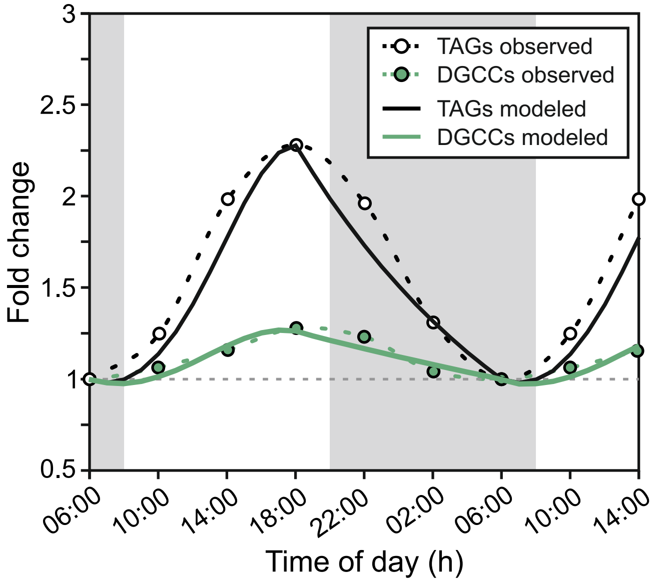

Supplementary Figure 11. Comparison between observed and modeled oscillations in lipid abundances. Shown are (1) time-of-day averages of fold change relative to 06:00 h (local time) of TAG and DGCC concentrations observed at 15 m water depth in July and August 2015 at Station ALOHA (white and green dots with dashed lines) and (2) modeled changes obtained using the inverse approach described in the Supplementary Methods (solid lines). TAG, triacylglycerol; DGCC, diacylglycerylcarboxy-*N*-hydroxymethyl-choline.

Supplementary Tables

Supplementary Table 1. Pfam domains associated with lipid metabolism identified in the North Pacific Subtropical Gyre metatranscriptome. Additionally shown are transcript abundances and diel periodicity for the five major taxonomic groups.

PFAM ID	PFAM name	Description	Taxonomic group	Mean transcript abundance (counts x 10^5 L^{-1})[a]	Diel periodicity[b]	Peak time (h)
PF01553	Acyltransferase	Acyltransferase	Haptophyceae	2.53±0.95	Yes	600
			Dinophyceae	19.4±4.12	No	-
			Pelagophyceae	0.28±0.099	No	-
			Bacillariophyta	0.49±0.19	No	-
			Chlorophyta	0.22±0.11	No	-
			Other	3.5±0.78	No	-
PF01764	Lipase_3	Triglyceride lipase	Haptophyceae	14.48±3.44	No	-
			Dinophyceae	131.6±25.35	No	-
			Pelagophyceae	1.56±0.35	No	-
			Bacillariophyta	8.03±1.57	No	-
			Chlorophyta	3.51±0.7	No	-
			Other	25.11±4.2	No	-
PF06762	LMF1	Lipase maturation factor	Haptophyceae	0.42±0.14	No	-
			Dinophyceae	5.0±1.21	No	-
			Pelagophyceae	0.11±0.08	No	-
			Bacillariophyta	0.93±0.31	No	-
			Chlorophyta	0.22±0.09	No	-
			Other	1.58±0.43	No	-
PF00887	ACBP	Acyl-CoA-binding protein	Haptophyceae	2.87±0.69	Yes	1800
			Dinophyceae	30.32±5.93	No	-
			Pelagophyceae	0.72±0.19	No	-
			Bacillariophyta	1.05±0.32	No	-
			Chlorophyta	3.01±0.49	No	-
			Other	20.36±3.33	No	-

[a]Transcript abundances represent average values ± SD over the sampling period (n = 48). [b]Diel periodicity of transcripts within taxonomic bins for the whole gene family was tested using the RAIN package in R[8].

Supplementary Table 2. Definition and values of optimized model parameters for the estimation of TAG loss at night due to cellular respiration.

Term	Symbol	Value	Units
Growth yield	φ_g	0.0619	$(h \times \text{arbitrary } I)^{-1}$
TAG yield	φ_s	0.0825	$(h \times \text{arbitrary } I)^{-1}$
Mortality rate	m	0.0192	h^{-1}
Night respiration factor	r	0.0473	h^{-1}

Supplementary References

1. Sarmiento, J. L. *et al.* Response of ocean ecosystems to climate warming. *Global Biogeochem. Cycles* **18,** GB3003 (2004).

2. Platt, T. & Irwin, B. Caloric content of phytoplankton. *Limnol. Oceanogr.* **18,** 306–310 (1973).

3. Buesseler, K. O. *et al.* Revisiting Carbon Flux Through the Ocean's Twilight Zone. *Science* **316,** 567 LP-570 (2007).

4. Steinberg, D. K. *et al.* Bacterial vs. zooplankton control of sinking particle flux in the ocean's twilight zone. *Limnol. Oceanogr.* **53,** 1327–1338 (2008).

5. Carini, P. *et al.* SAR11 lipid renovation in response to phosphate starvation. *Proc. Natl. Acad. Sci.* **112,** 7767–7772 (2015).

6. Elling, F. J. *et al.* Respiratory quinones in Archaea: phylogenetic distribution and application as biomarkers in the marine environment. *Environ. Microbiol.* **18,** 692–707 (2016).

7. Matsen, F. A., Kodner, R. B. & Armbrust, E. V. pplacer: linear time maximum-likelihood and Bayesian phylogenetic placement of sequences onto a fixed reference tree. *BMC Bioinformatics* **11,** 538 (2010).

8. Thaben, P. F. & Westermark, P. O. Detecting Rhythms in Time Series with RAIN. J. Biol. Rhythms 29, 391–400 (2014).
