# Supplementary material for: Daily changes in phytoplankton lipidomes reveal mechanisms of energy storage in the open ocean
Source: Nat Commun. 2018 Dec 5;9:5179. doi: 10.1038/s41467-018-07346-z (PMC6281602; doi:10.1038/s41467-018-07346-z)
Supplement: Supplementary file 3 — Description of Additional Supplementary Files [file 41467_2018_7346_MOESM3_ESM.pdf]

Description of Additional Supplementary Files

File Name: Supplementary Data 1
Description: Identified lipids using the LOBSTAHS processing pipeline. Lipids are identified by the "sum composition" of double bonds and acyl carbon atoms in each compound (e.g., DGCC-C32:1, rather than DGCC-C16:0−16:1). Also given are the measured mass, the calculated accurate mass and their offset in ppm.
